# 739. Self-Reported Prevalence of Insect Bites During International Travel

**DOI:** 10.1093/ofid/ofab466.936

**Published:** 2021-12-04

**Authors:** Holly Shoemaker, Michael Graves, Sharia Ahmed, Holly K Birich, Scott Benson, John R Contreras, Colette McAfee, Daniel T Leung

**Affiliations:** 1 University of Utah School of Medicine, Salt Lake City, Utah; 2 University of Utah Division of Infectious Diseases, West Valley City, Utah; 3 University of Utah, Salt Lake City, Utah; 4 Salt Lake County Health Department, Salt Lake City, Utah; 5 Westminster College, Salt Lake City, Utah

## Abstract

**Background:**

Vector borne diseases are responsible for almost one fifth of global infectious disease burden. International travelers are at risk for potentially life-threatening conditions when visiting areas with endemic vector borne disease, but this risk can be mitigated when proper insect precautions are taken. This study sought to evaluate the prevalence of insect precaution use and subsequent insect bites among Utah travelers who have attended pre-travel consultations.

**Methods:**

A cross-sectional study at the University of Utah and Salt Lake County travel clinics was analyzed. Descriptive statistics and multivariable logistic regression were used to explore factors associated with insect repellant use, and reporting bug bites despite insect repellant use.

**Results:**

A total of 463 individuals completed the survey and were included in our analytic sample. The majority of respondents (80%) reported using insect repellent, and close to half (45%) reported bug bites. Insect repellent use was positively associated with visiting rural/countryside (OR 2.78, 95% CI 1.50 – 5.15), and traveling to South East Asia (OR 3.16, 95% CI 1.40 – 7.26), or Americas regions (OR 3.34, 95% CI 1.45 – 7.92). Being of male gender (OR 0.37, 95% CI 0.21 – 0.64) or traveling to high altitude locations (OR 0.37, 95% CI 0.18 – 0.74) was negatively associated with using insect repellent. Longer trip duration (OR 1.01, 95% CI 1.00 – 1.02) was positively associated with reporting insect bites, while male gender (OR 0.51, 95% CI 0.33 – 0.80), older age (OR 0.96, 95% CI 0.95 – 0.98), and having an advanced degree (OR 0.47, 95% CI 0.22 – 0.99) were negatively associated.

Estimated Risk Factors of Insect Bites and Insect Repellent Use

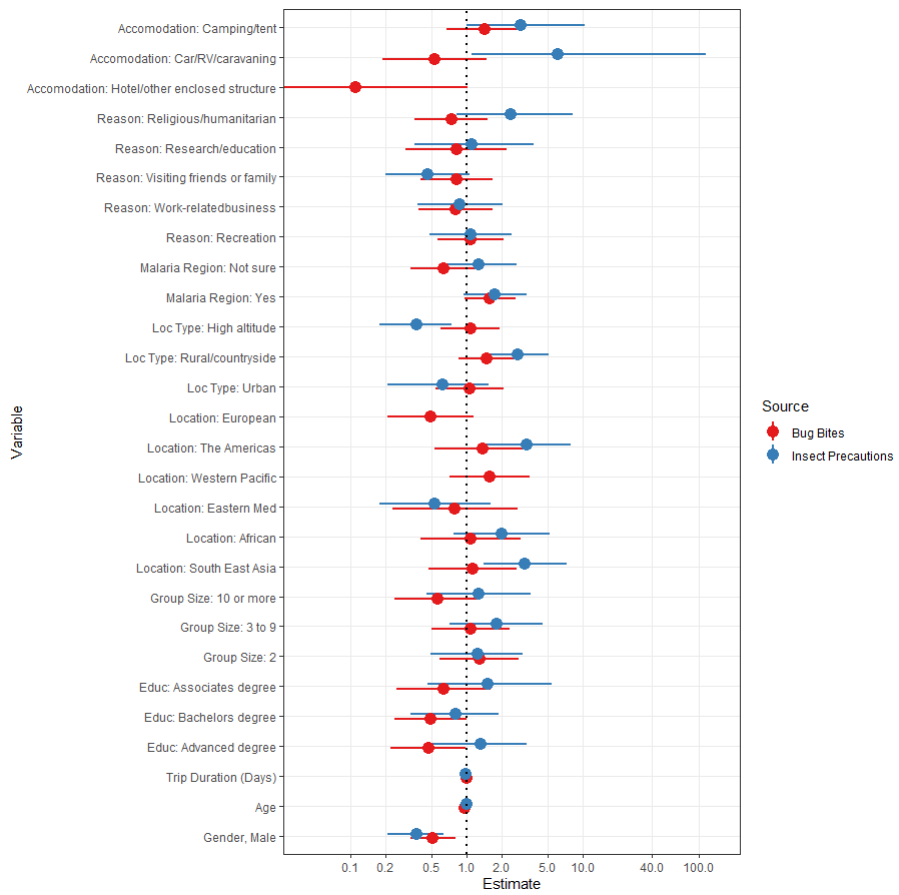

Characteristics of international travelers were self-reported in a cross-sectional study. Use of insect repellent and reporting bug bites despite repellant use was examined through multivariate logistic regression and used to calculate odds ratios and 95% confidence intervals. Due to multicollinearity and data skewness, the following variables were omitted from the insect repellent model: Accommodation: Hotel/other enclosed structure, Location: European, Location: and Western Pacific. Reference categories are Gender: Female, Education: High school diploma/GED or less, Group size: 1 (Traveled alone), Location type: Urban, and Malaria region: No. All other categories are not mutually exclusive and evaluated as separate binary variables.

**Conclusion:**

We show that gender, age, trip duration, and education level were associated with self-reported bug bites during travel abroad. Given the number of vector-borne diseases affecting health of travelers, our findings will contribute towards strategies to advise travelers for disease prevention.

**Disclosures:**

**All Authors**: No reported disclosures

